# Representation theory of coherent photons and application to CNOT operation for spin and orbital angular momentum

**DOI:** 10.1038/s41598-025-23755-9

**Published:** 2025-11-14

**Authors:** Shinichi Saito

**Affiliations:** Research & Development Group, Hitachi, Ltd., Tokyo, 185-8601 Japan

**Keywords:** Quantum optics, Micro-optics, Micro-optics, Qubits

## Abstract

Coherent photons have both spin and orbital angular momentum, and the superposition state among orthogonal states can describe a qubit. Here, we show the internal structure of coherent photons with spin and orbital angular momentum can be described by a representation theory of Lie algebra and Lie group. As an application of this theory, we have experimentally demonstrated the controlled-NOT (CNOT) operation, using a standard green laser diode. We have used beam splitters and waveplates to generate the macroscopic entangled light source with amplitudes and phases, controlled by rotating the waveplates. By applying the Bell projection to the entangled light, the far-field image of the light shows an expected dipole shape in the superposition state of left and right vortices, allowing to identify the state from the image. Then, we have used a polarisation dependent beam splitter to split the spin state, and we applied a NOT operation, made of a pair of cylindrical lenses, only for a vortex with a chosen polarisation, and recombined the beams after the operation. The proposed architecture could be used for a platform to manipulate qubits, made of macroscopically coherent photons.

## Introduction

One of the most intriguing motivation to develop quantum technologies is to understand why our macroscopic world is governed by classical-mechanics, even though the fundamental law of physics for its microscopic constituent is following to quantum-mechanics^1,2^. Most of macroscopic matters cannot behave like an elementary particle, which can be a superposition state among orthogonal states, as famous for a paradox of Schrödinger’s cat^1,2^. However, there are a few exceptional systems, where quantum coherence is observed in a macroscopic scale, such as superconductivity^3^, Bose-Einstein Condensation (BEC)^4^, and a laser^5–7^. In these systems, continuous symmetry is spontaneously broken upon onsets of phase transitions^3^ or pumping above lasing thresholds, and the entire systems are described by sole wavefunctions. For coherent photons emitted from widely available laser sources, however, it is generally believed that the polarisation state is classical, characterised by Stokes parameters^5^ on a topologically trivial Poincaré sphere^5^, while these parameters are calculated quantum-mechanically using the two-level systems with the special unitary group of degree two, known as SU(2)^6–9^. On the other hand, there are growing evidences that the so-called classical light can behave quantum mechanically including the entanglement^8–18^.

Here, we show that this apparent contradiction on polarisation whether it is classical or quantum-mechanical is solved by employing a quantum many-body theory together with a representation theory of Lie algebra and Lie group^19–23^. The SU(2) symmetry is not restricted to the spin state of photons as polarisation, but it can also be applied to include orbital angular momentum^13,14,24–39^. We show that coherent photons are described by a macroscopic wavefunction of SU(*N*) states with spin and orbital angular momentum as internal degrees of freedom, where the degree of *N* is determined by the dimension of orthogonal basis states. Based on the SU(*N*) theory for coherent photons, we have confirmed that the phases and the amplitudes of the macroscopic wavefunction could be controlled by combinations of standard optical components, and the expectation values for generators of rotations were observed as Stokes and even higher order parameters. This could serve as a theoretical basis to use structured lights for various quantum technologies^8,9,11–17,40^. As an application of the theory, we have experimentally demonstrated the controlled-NOT (CNOT) operation for coherent photons with spin and orbital angular momentum. The purpose of this paper is to explore analogies and differences between quantum and classical entanglement for potential application to quantum technologies.

## Theory

We consider a ray of coherent photons with SU(*N*) symmetry for angular momentum, which is given by the wavefunction for a coherent state^3,41,42^ ,

1 $$\begin{aligned} |\alpha _1, \cdots , \alpha _N \rangle= & \prod _{\sigma =1}^{N} \textrm{e}^{-\frac{|\alpha _\sigma |^2}{2}} \textrm{e}^{ {\hat{a}}_\sigma ^{\dagger } \alpha _\sigma } |0 \rangle , \end{aligned}$$

where ${\hat{a}}_\sigma ^{\dagger }$ (${\hat{a}}_\sigma$) is a photon creation (annihilation) operator, which satisfies the Bose commutation relationship of $[{\hat{a}}_\sigma , {\hat{a}}_{\sigma ^{\prime }}^{ \dagger }]=\delta _{\sigma ,\sigma ^{\prime }}$, $\sigma$ stands for the $\sigma$-th component of the SU(*N*) degrees of freedom for spin and orbital angular momentum, and $\alpha _\sigma$ is a complex number (${\mathbb {C}}$) to represent the macroscopic wavefunction. The SU(*N*) degrees of freedom is related to the rotational symmetry among *N* orthogonal states in the Hilbert space, whose rotation is given by the exponential map of the ${{\mathfrak {s}}}{{\mathfrak {u}}}(N)$ Lie algebra,

2 $$\begin{aligned} {{\mathcal {D}}}_i (\theta )= & \textrm{e}^{-i {X}_i \frac{\theta }{2}}, \end{aligned}$$

where ${X}_i$ is the generator of rotation, made of a complex matrix of $N \times N$, which satisfies the commutation relationship of $[ {X}_i, {X}_j ] = 2i \sum _{k} f_{i j k} {X}_k$, with the structure constant of $f_{i j k}$, for the amount of the rotation, $\theta$, along the *i*-th axis. In general, there are $(N^2 - 1)$ generators for ${{\mathfrak {s}}}{{\mathfrak {u}}}(N)$, such that $i, j, k=1, \cdots , (N^2 - 1)$. ${{\mathcal {D}}}_i (\theta )$ is also a complex matrix of $N \times N$, which transfers ${\hat{a}}_\sigma ^{\dagger }$ to $\sum _{\sigma ^{\prime }} {\hat{a}}_{\sigma ^{\prime }}^{\dagger } {{\mathcal {D}}}_{i \sigma ^{\prime } \sigma } (\theta )$, upon the rotation, or equivalently, it changes the macroscopic wavefunction in the initial state, $|\textrm{I} \rangle = (\alpha _1, \cdots , \alpha _N)^{\textrm{t}}$ to the final state $|\textrm{F} \rangle = {{\mathcal {D}}}_i (\theta ) |\textrm{I} \rangle$, where $^{\textrm{t}}$ stands for the transpose.

For spin angular momentum of coherent photons, we use SU(2) states for horizontal-vertical (HV) linearly-polarised or left-right (LR) circularly-polarised states as fundamental bases^5–7^, and the generators of rotations are given by Pauli matrices, ${\sigma }_i$ ($i=1,2,3$), which satisfy $[ {\sigma }_i, {\sigma }_j ] = 2 i \sum _{k} \epsilon _{i j k} {\sigma }_k$, where $\epsilon _{i j k}$ is the totally asymmetric tensor. We have obtained the spin angular momentum operator in the many-body state, as $\hat{\bf{S}}=\hbar \hat{\boldsymbol{\psi }}^{\dagger } {\boldsymbol{\sigma }} \hat{\boldsymbol{\psi }}$, where $\hat{\boldsymbol{\psi }}^{\dagger }=({\hat{a}}_{\textrm{H}}^{\dagger } , {\hat{a}}_{\textrm{V}}^{\dagger } )$ and $\hat{\boldsymbol{\psi }}=({\hat{a}}_{\textrm{H}}, {\hat{a}}_{\textrm{V}} )^{\textrm{t}}$ are the spinor representation of creation and annihilation operators in HV bases to create and annihilate a photon, and ${\boldsymbol{\sigma }} = (\sigma _3, \sigma _1, \sigma _2)$ are generators of spin angular momentum. In the HV bases, the macroscopic wavefunction is given by ${\boldsymbol{\alpha }}=(\alpha _{\textrm{H}},\alpha _{\textrm{V}})^{\textrm{t}}=\sqrt{{\mathcal {N}}}(\cos \alpha , \sin \alpha \textrm{e}^{i \delta })^{\textrm{t}}$, where ${{\mathcal {N}}}$ is the average density of photons passing through the cross sectional area per second along the direction of the propagation , $\alpha$ is the auxiliary angle, and $\delta$ is the phase. Then, it is straightforward to calculate the quantum-mechanical average of $\hat{\bf{S}}$, since the coherent state is the eigenstate of $\hat{\boldsymbol{\psi }}$, and we obtain $\langle \hat{\bf{S}} \rangle _{\textrm{I}}= \hbar {{\mathcal {N}}}(\cos (2\alpha ), \sin (2\alpha ) \cos \delta , \sin (2\alpha ) \sin \delta )$, where $\hbar$ is the Dirac constant. $\langle \hat{\bf{S}} \rangle _{\textrm{I}}$ is equivalent to normalised Stokes parameters of ${\bf{S}}=(S_1, S_2, S_3)$, but we also obtained the overall magnitude, $S_0=\hbar {{\mathcal {N}}}$, which means the Plank constant ($h=2 \pi \hbar$) effectively becomes macroscopic for coherent photons.

Moreover, above relationship between SU(*N*) states and generators of rotation leads to a hyperspherical formula for expectation values in the final state upon the rotation of $\theta$ as

3 $$\begin{aligned} \langle {X}_j \rangle _{\textrm{F}} = \sum _k \left( \textrm{e}^{- {F}_{i} \theta } \right) _{jk} \langle {X}_k \rangle _{\textrm{I}}, \end{aligned}$$

where ${F}_{i}$ is an adjoint operator, whose matrix element becomes $({F}_{i})_{jk}=f_{ijk}$, which is a matrix of $(N^2-1)\times (N^2-1)$. This means that the rotation of the wavefunction in the Hilbert space of SU(*N*) corresponds to the rotation of expectation values in the special orthogonal group of SO($N^2-1$). For spin angular momentum of SU(2)^5–7^, we obtain $f_{ijk}=\epsilon _{ijk}$, which corresponds to the rotation of Stokes parameters on the Poincaré sphere in SO(3)^43^.

Now, we understand why Stokes parameters on Poincaré sphere can describe states for macroscopic number of coherent photons emitted from a laser. The original rotational symmetry of the system is broken upon the lasing threshold, and the single mode (or a few modes, depending on the quality of the cavity) is naturally selected upon stimulated emissions. Thus, the whole system is described by a sole macroscopic wavefunction, which has internal degrees of freedom for spin angular momentum, and the existence of the Nambu-Anderson-Higgs-Goldstone mode upon symmetry-breaking guarantees that we can still rotate the whole wavefunction without spending energies^44^. Consequently, we can manipulate the polarisation state by using wave-plates and phase-shifters, which work as quantum-mechanical rotation operators in SU(2), ${{\mathcal {D}}}_i^{\textrm{S}}(\theta )=\textrm{e}^{-i {\sigma }_i \theta /2}$. We also understand the inherent relationship between angular momentum states^5–7^ and a representation theory^17,19–23^.

**Fig. 1:**
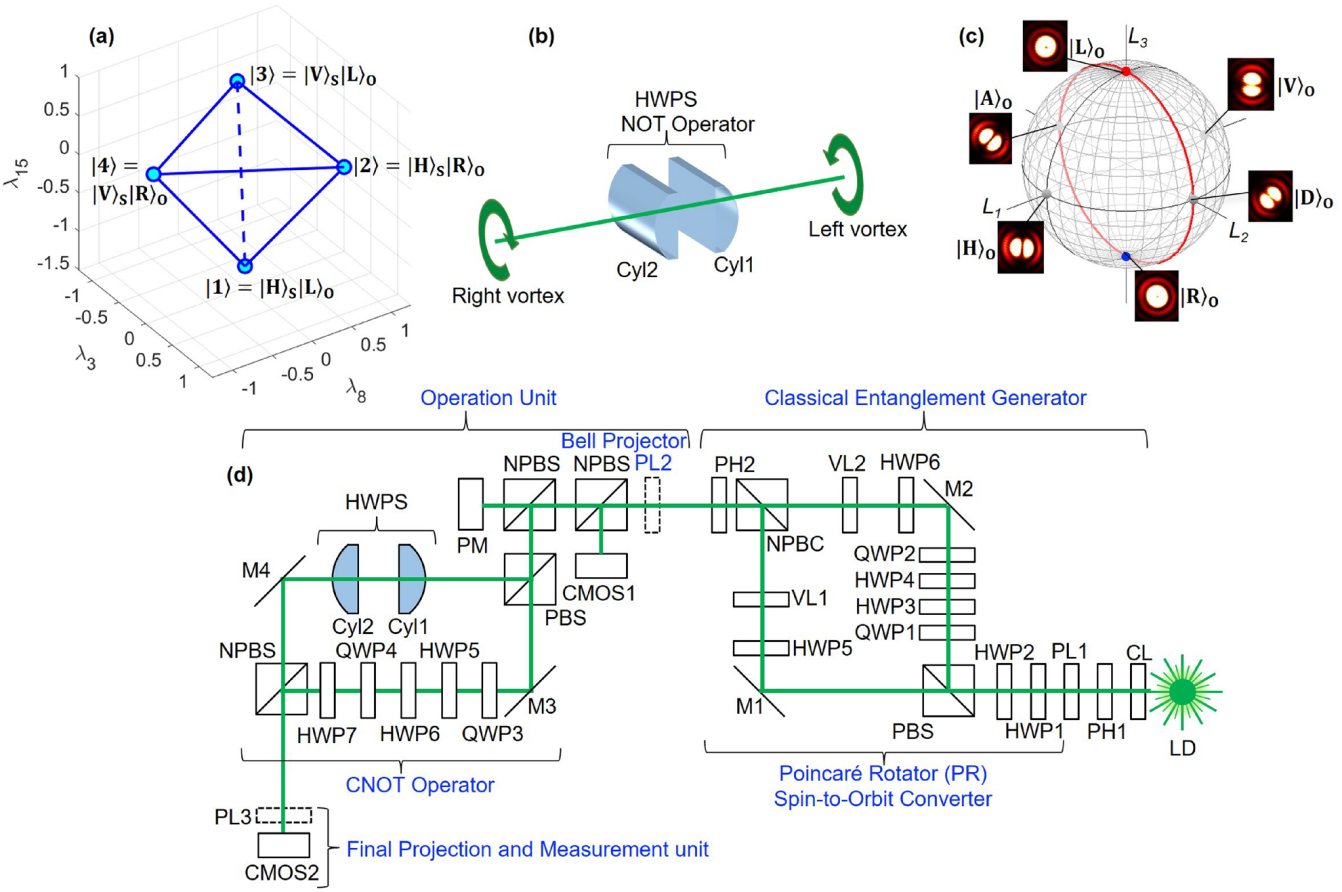
CNOT operation for coherent photons. (**a**) The weight diagram of SU(4) states of coherent photons with spin and orbital angular momentum. (**b**) NOT operator for orbital angular momentum. Two cylindrical lenses are separated with the twice of the focal length to achieve the phase-shift of $\pi$ for converting the left vortex to the right vortex, and *vice versa*. (**c**) Poincaré sphere for orbital angular momentum. Far-field images are shown for left (L) and right (R) twisted states and their superposition states of horizontal (H), diagonal (D), vertical (V), and anti-diagonal (A) dipole states, respectively. The red circle shows how the half-wave phase-shift of (b) changes the twisted states. (**d**) Experimental set-up for CNOT operation to coherent photons with spin and orbital angular momentum. The system is made of three units, a classical entanglement generator, an operation unit, and a measurement unit. The generator is made of a Poincaré rotator, which allows the arbitrary rotation of polarisation states, and vortex lenses to allow spin-to-orbit converter. The generated entangled light is subject to the optional Bell projection, to allow changes in orbital angular momentum states by projection of spin state. The CNOT operation is achieved by splitting the spin state by a polarisation dependent beam splitter and apply the NOT operation to vertically polarised beam, while the horizontally polarised beam is preserved, and then recombined. *Cyl* cylindrical lens, *HWPS* half-wave phase-shifter, *LD* laser diode, *CL* collimator lens, *PH* pin hole, *PL* polariser, *HWP* half-wave plate, *QWP* quarter-wave plate, *PBS* polarisation beam splitter, *NPBC* non-polarisation beam combiner, *NPBS* non-polarisation beam splitter, *M* mirror, *VL* vortex lens, *PM* polarimeter, *CMOS* camera. PL2 and PL3 are optional and shown by the dotted lines.

The representation theory for coherent photons can be readily applicable to orbital angular momentum^13,14,24–28,30–38^. We consider both left (L) and right (R) vortices with the topological charge of 1. There are four orthogonal states ($N=4$) of spin and orbital angular momentum as $|1\rangle = |\textrm{H}\rangle _{\textrm{S}}|\textrm{L}\rangle _{\textrm{O}}$, $|2\rangle = |\textrm{H}\rangle _{\textrm{S}}|\textrm{R}\rangle _{\textrm{O}}$, $|3\rangle = |\textrm{V}\rangle _{\textrm{S}}|\textrm{L}\rangle _{\textrm{O}}$, and $|4\rangle = |\textrm{V}\rangle _{\textrm{S}}|\textrm{R}\rangle _{\textrm{O}}$, where the subscripts of S and O stands for spin and orbital angular momentum states, respectively. The weight diagram^19–23^ to represent these four states in SU(4) is shown in Fig. 1 (a). The states are shown at the apexes of the tetrahedron, which mean that these four states are equally important and separated in equal distance. For describing the rotation of these states, we use $4\times 4$ matrices, while one of the component is determined by the traceless condition for ${{\mathfrak {s}}}{{\mathfrak {u}}}(4)$ Lie algebra^19–23^. Therefore, we have $4^2-1=15$ generators of rotation in ${{\mathfrak {s}}}{{\mathfrak {u}}}(4)$, and three of them, $\lambda _3$, $\lambda _8$, and $\lambda _{15}$ are diagonal and the weight diagram is described by these quantum numbers^19–23^. We can construct arbitrary SU(2) states by picking up two states among four states. For example, by considering the coupling between $|1\rangle$ and $|3\rangle$ (or, between $|2\rangle$ and $|4\rangle$), we can realise an arbitrary polarisation state for a left vortex (a right vortex). Similarly, by coupling between $|1\rangle$ and $|3\rangle$ ($|2\rangle$ and $|4\rangle$), we can realise an arbitrary twisted state for left (right) polarisation for the average orbital angular momentum of ${\bf{L}}=(L_1, L_2, L_3)$^25^ (Fig. 1 (c)). We have summarised the single-qubit operations in Table 1. We can apply SU(4) operation for coherent photons exclusively for the spin (orbital) angular momentum state without changing the orbital (spin) angular momentum state. For example, if we apply ${\bf{1}} \otimes \sigma _1$ to coherent photons, where ${\bf{1}}$ is the identity operator in SU(2), we will exchange left and right orbital angular momentum, while keeping polarisation, and the overall operation is the NOT operation for orbital angular momentum. There are three operators for spin, $\sigma _1 \otimes {\bf{1}}$, $\sigma _2 \otimes {\bf{1}}$, and $\sigma _3 \otimes {\bf{1}}$. Similarly, three orbital angular momentum operators are ${\bf{1}} \otimes \sigma _1$, ${\bf{1}} \otimes \sigma _2$, and ${\bf{1}} \otimes \sigma _3$ (Table 1).

**Table 1:**
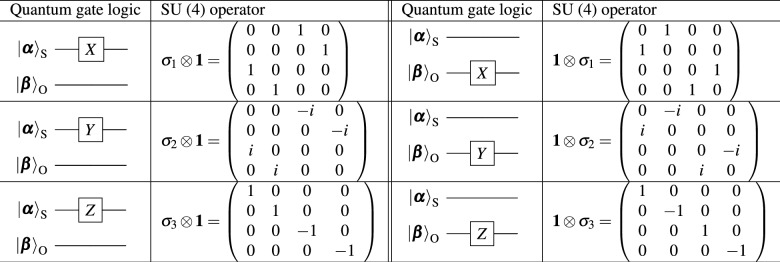
Single-qubit operation for SU(4) states of coherent photons for the spin angular momentum state of $|{\boldsymbol{\alpha }} \rangle _{\textrm{S}}$ and the orbital angular momentum state of $|{\boldsymbol{\beta }} \rangle _{\textrm{O}}$. The identity operator of ${\bf{1}}$ stands for the unit operation for SU(2) states.

In addition to above six single-qubit operators, we have nine two-qubit operators, $\sigma _i \otimes \sigma _j$ ($i,j=1,2,3$) for couplings between singlet and triplet states to cover all fifteen generators of rotations in ${{\mathfrak {s}}}{{\mathfrak {u}}}(4)$. The singlet/triplet states correspond to the coupling between $|2\rangle$ and $|3\rangle$, which is famous for the higher order Poincaré sphere^30,45,46^, and we can also consider the triplet/triplet coupling between $|1\rangle$ and $|4\rangle$. These six ways of SU(2) couplings show how simple SU(4) states with spin and orbital angular momentum can represent varieties of states, and we can also consider the complex mixing of all these four states with variable amplitudes and phases. It is a tremendous challenge to prepare an arbitrary rotational operator in SU(4), but fortunately, it was shown that CNOT operation together with the single-qubit operations are enough to realise an arbitrary two-qubit operations^36,40,47^. Next, we show how we could achieve CNOT operation for coherent photons.

In order to realise the CNOT operation, we employ the ${{\mathfrak {s}}}{{\mathfrak {u}}}(4)$ operator of ${\lambda }_{13}$, which is defined as

4 $$\begin{aligned} {\lambda }_{13}= & \frac{1}{2} ({\bf{1}} - \sigma _3) \otimes \sigma _1 = \left( \begin{array}{cccc} 0 & 0 & 0 & 0 \\ 0 & 0 & 0 & 0 \\ 0 & 0 & 0 & 1 \\ 0 & 0 & 1 & 0 \end{array} \right) , \end{aligned}$$

where the summation and the multiplication of the real number (${{\mathbb {R}}}$) are allowed in ${{\mathfrak {s}}}{{\mathfrak {u}}}(4)$, since the Lie algebra is a vector space^21–23^. Then, the corresponding SU(4) operator is obtained as an exponential map of ${{\mathcal {D}}}_{13}(\pi ) = \textrm{e}^{-i {\lambda }_{13} \pi /2}$. Together with the phase control of ${{\mathcal {D}}}_3^{\textrm{S}}(\pi /2)=\textrm{e}^{-i {\sigma }_3 \pi /4}$, we obtain the CNOT operator,

5 $$\begin{aligned} {{\mathcal {D}}}_{\textrm{CNOT}}= & \textrm{e}^{i \pi /4} {{\mathcal {D}}}_{13}(\pi ) {{\mathcal {D}}}_3^{\textrm{S}}(\pi /2) \end{aligned}$$

6 $$\begin{aligned}= & \textrm{e}^{i \pi /4} \left( \begin{array}{cccc} 1 & 0 & 0 & 0 \\ 0 & 1 & 0 & 0 \\ 0 & 0 & 0 & -i \\ 0 & 0 & -i & 0 \end{array} \right) \left( \begin{array}{cccc} 1 & 0 & 0 & 0 \\ 0 & 1 & 0 & 0 \\ 0 & 0 & i & 0 \\ 0 & 0 & 0 & i \end{array} \right) \end{aligned}$$

7 $$\begin{aligned}= & \left( \begin{array}{cccc} 1 & 0 & 0 & 0 \\ 0 & 1 & 0 & 0 \\ 0 & 0 & 0 & 1 \\ 0 & 0 & 1 & 0 \end{array} \right) , \end{aligned}$$

where the overall global phase of $\textrm{e}^{i \pi /4}$ was inserted to follow the standard convention of the CNOT operator^40^. In table 2, we show the two-qubit operation for coherent photons represented by the SU(4) operator.

**Table 2:**
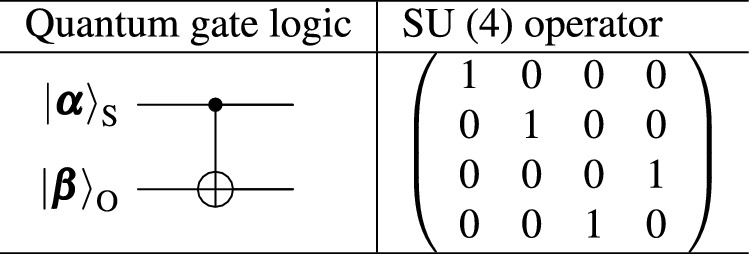
Two-qubit operation for SU(4) states of coherent photons for the spin angular momentum state of $|{\boldsymbol{\alpha }} \rangle _{\textrm{S}}$ and the orbital angular momentum state of $|{\boldsymbol{\beta }} \rangle _{\textrm{O}}$.

## Results

The experimental set-up is shown in Fig. 1b, d (Methods).

### NOT operation

Firstly, we have confirmed the NOT operation for orbital angular momentum (Fig. 1b). Here, we have connected the classical entanglement generator, directly to the pair of the cylindrical lenses (Cyl1 and Cyl2)^48^.

The cylindrical lenses were separated with the distance of 2*f*, where *f* is the focal length, such that the collimation was kept upon passing through the Half-Wave-Phase-Shifter (HWPS) for vortices. This allows us to convert the left vortex to the right vortex, and vice versa, which corresponds to the NOT operation, ${{\mathcal {D}}}_{\textrm{O}}(\pi )=-i \sigma _{1}^{\textrm{O}}$, for orbital angular momentum in LR bases, where the superscript of O stands for the operator application to orbital angular momentum states. $\sigma _{1}^{\textrm{O}}$ should work exclusively for the SU(2) states of orbital angular momentum. The factor of $-i$ was included to guarantee that SU(2) is the two-fold coverage for SO(3)^21–23^. We have set the PL2 to the diagonal direction to visualise the orbital angular momentum in the far-field images.

**Fig. 2:**
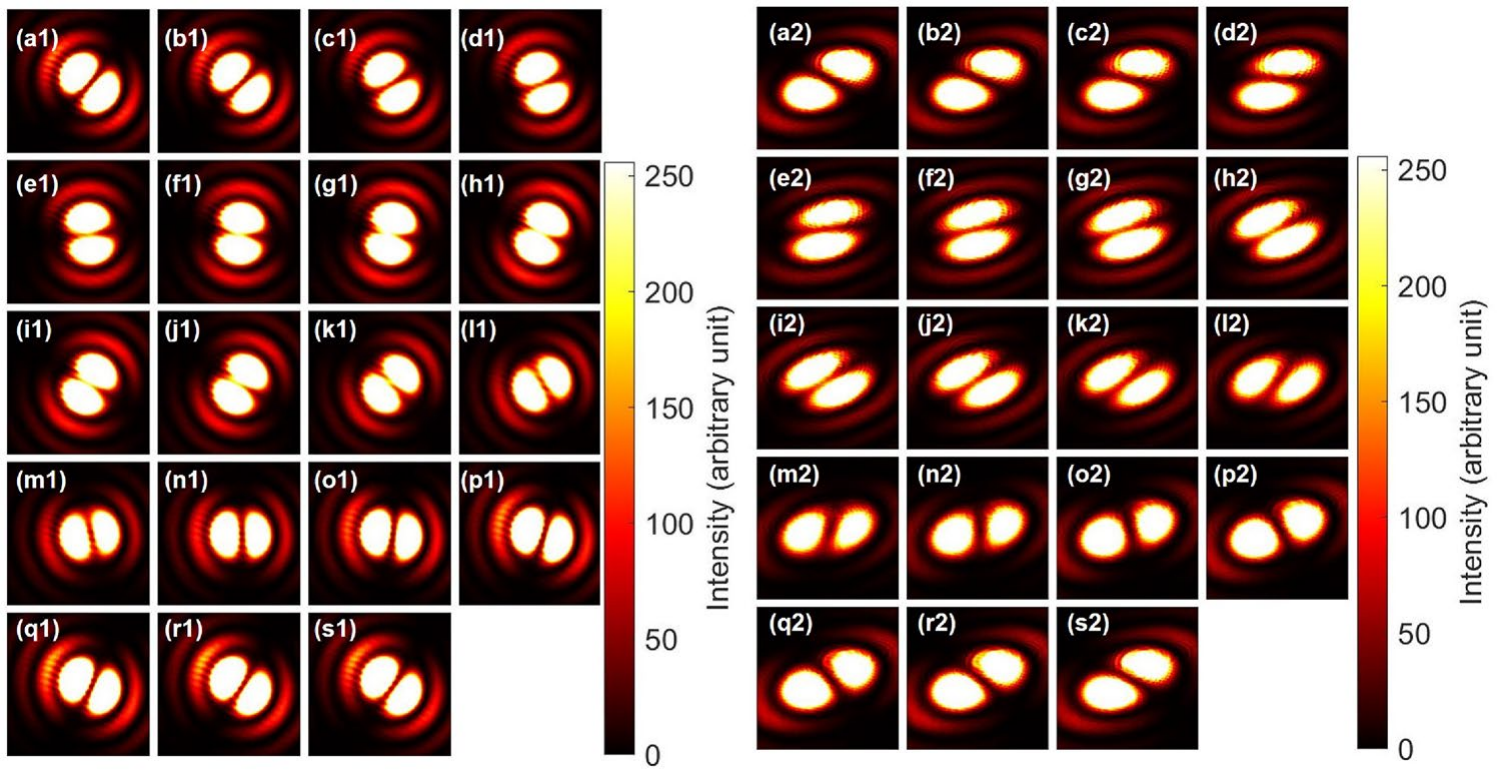
Phase-shifter rotation for twisted photons and NOT operation for the half-wave phase-shift in orbital angular momentum. (**a1**)–(**s1**) far-field images of input beam at CMOS1. HWP4 was rotated from 0 to 180 $^{\circ }$ with a step of 10 $^{\circ }$. PL2 was set to the diagonal direction. (**a2**)–(**s2**) The corresponding far-field images for the output after the NOT operation. The dipoles in the input images are rotating to the clock-wise direction, while the dipoles in the output images are rotating along the counter clock-wise direction.

Figure 2 shows typical NOT operations for orbital angular momentum states. The input beams were controlled by the phase-shifter, and we have rotated the half-wave-plate (HWP4) to rotate the dipole along the clock-wise direction (Fig. 2(a1)–(s1)). The corresponding NOT operations were shown in Fig. 2(a2)–(s2). Specifically, for example, the anti-diagonal input of Fig. 2(a1) was rotated to the orthogonal diagonal direction in Fig. 2(a2). The opposite was also true for the diagonal input of Fig. 2(j1) to the anti-diagonal output of Fig. 2 (j2). The output dipoles were rotated along the counter-clock-wise direction (Figs. 2(a2)–(s2)), which is opposite to the input. This could be understood that the NOT operation is the $\pi$ rotation along the $L_1$ axis, such that the direction of rotation will be reversed, while horizontal and vertical dipoles were preserved. In fact, the vertical dipole of Fig. 2(e1) and the horizontal dipole of Fig. 2(n1) were not affected significantly upon the NOT operations, as shown in Fig. 2(e2) and (n2), respectively.

**Fig. 3:**
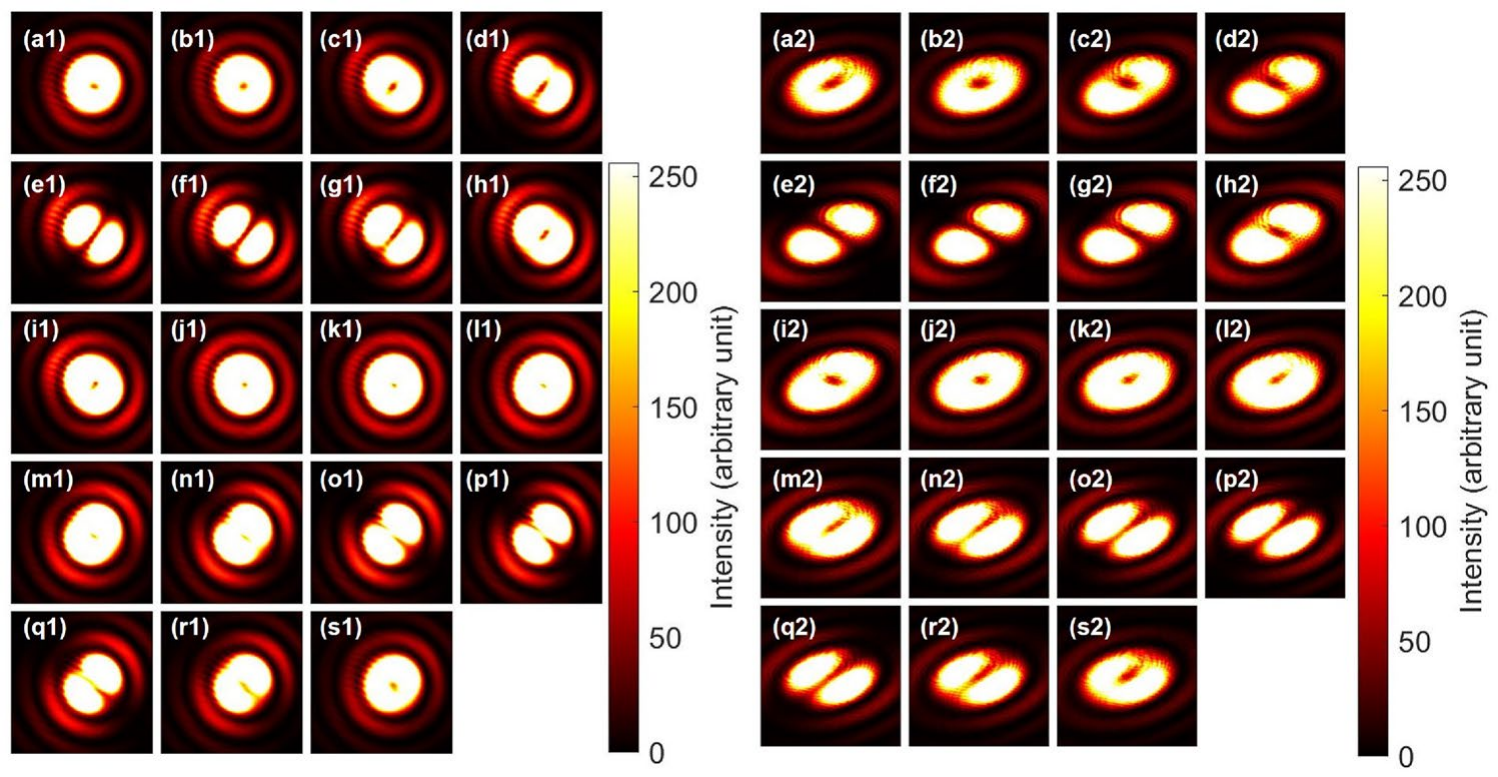
Rotator operation for twisted photons and NOT operation for the half-wave phase-shift in orbital angular momentum. (**a1**)–(**s1**) far-field images of input beam at CMOS1. HWP2 was rotated from 0 to 90 $^{\circ }$ with a step of 5 $^{\circ }$. PL2 was set to the diagonal direction. The phase was adjusted by HWP4 to form a singlet at the image of (e1), where the dipole is anti-diagonally aligned, orthogonal to the diagonal polarisation. (**a2**)–(**s2**) The corresponding far-field images for the output after the NOT operation. The rotator operation corresponds to change the relative amplitudes between left and right vortices for the input, which corresponds to rotate along the longitude, shown as the red line in Fig. 1c. The CNOT operation corresponds to the additional $\pi$ rotation along the same circulation, converting the left to the right vortices and vice versa, and converting the diagonal dipole to the anti-diagonal dipole and vice versa.

We have also used the rotator operation for changing the input, as shown in Fig. 3. Here, we have rotated HWP2 to change the relative amplitudes for left and right vorticies. The input orbital angular momentum states were changed from Fig. 3(a1) the right vortex to (e1) the anti-diagonal dipole, (j1) the left vortex, (o1) the diagonal dipole, and back to the (s1) the right vortex. The corresponding outputs after the NOT operation were Fig. 3(a2) the left vortex to (e2) the diagonal dipole, (j2) the right vortex, (o2) the anti-diagonal dipole, and back to the (s1) the left vortex. It is not possible to distinguish the left vortex with the right vortex, however, we can confirm the diagonal dipole was successfully converted to the anti-diagonal dipole, and the vice versa. Therefore, the NOT operation was applied, as expected.

**Fig. 4:**
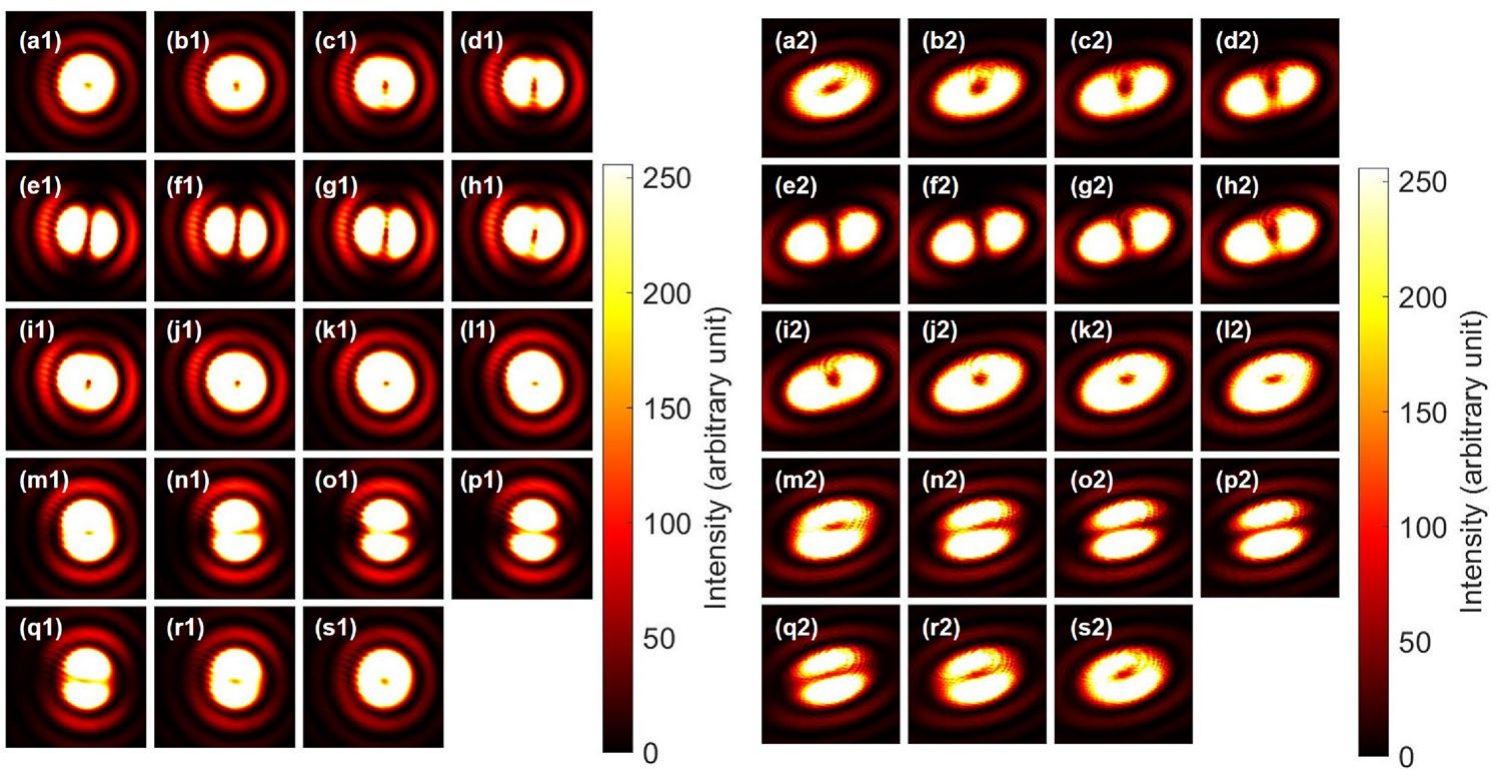
Rotator operation for twisted photons and NOT operation for the half-wave phase-shift in orbital angular momentum. (**a1**)–(**s1**) far-field images of input beam at CMOS1. HWP2 was rotated from 0 to 90 $^{\circ }$ with a step of 5 $^{\circ }$. PL2 was set to the diagonal direction. The phase was adjusted by HWP4 to align the dipole to point along the horizontal direction at the image of (**e1**). (**a2**)–(**s2**) The corresponding far-field images for the output after the NOT operation. The rotator operation corresponds to change the orbital angular momentum state from the left vortex to the horizontal dipole, the right vortex, the vertical vortex, and the come back to the left vortex. This corresponds to the counter-clock-wise rotation along the $L_2$ axis. The CNOT operation corresponds to the $\pi$ rotation along the $L_1$ axis, which preserves the horizontal (**e2**) and the vertical (**o2**) dipoles unchanged.

Furthermore, we have also adjusted to the phase by HWP4 that the input dipole is aligned to the horizontal direction, as shown in Fig. 4(e1). In this case, the horizontal and vertical dipoles of the inputs in Figs. 2 (e1) and (n1) have not been changed upon the NOT operation, as shown in Figs. 2(e2) and (n2), respectively. This is expected from the $\pi$ rotation along the $L_1$ axis, such that the rotator operation of Fig. 4 is consistent with the above phase-shifter operation of Fig. 2.

**Fig. 5:**
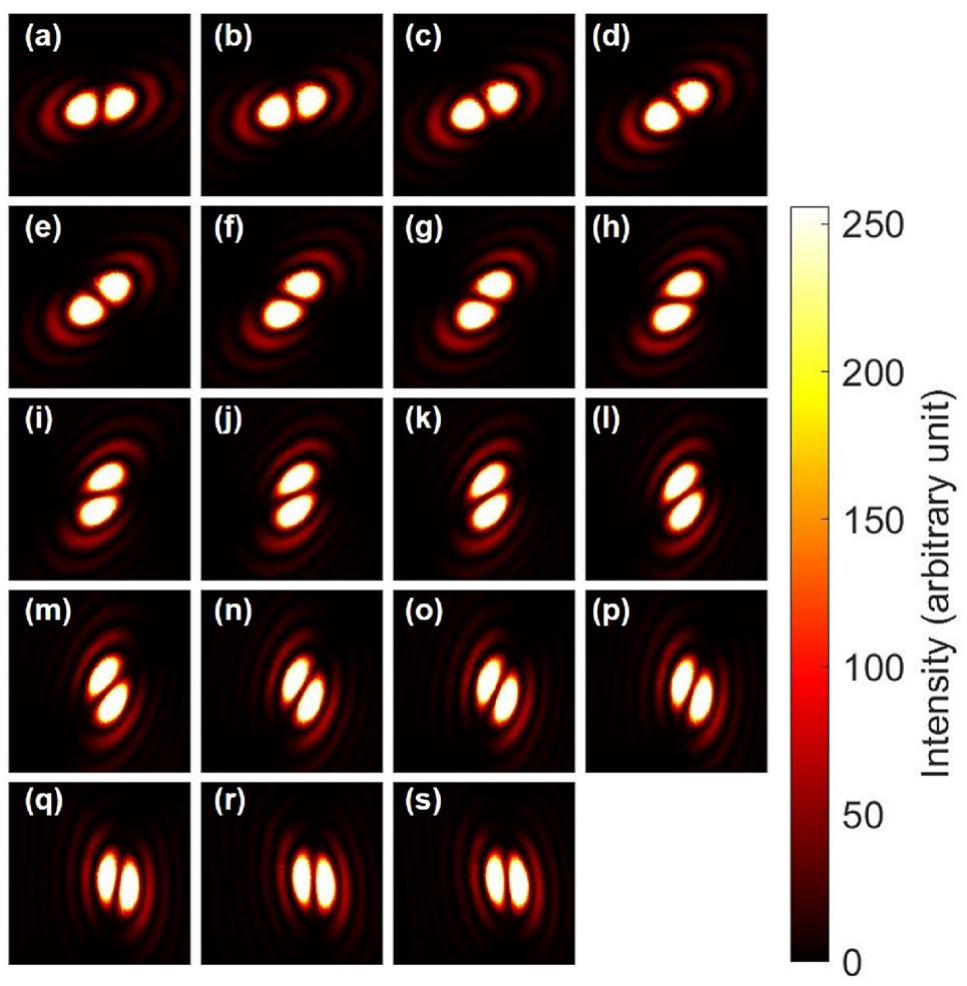
Pseudo-rotator operation of the rotated pair of cylindrical lenses. (**a**)–(**s**) The pair of cylindrical lenses was rotated from 0 to 90 $^{\circ }$ with a step of 5 $^{\circ }$, while keeping the relative faces with the fixed separation distance of 2*f*. The dipole rotated along the counter-clock-wise direction from (**a**) horizontal to (**e**) diagonal, (**j**) vertical, (**o**) anti-diagonal, and back to the horizontal directions. Please note that the phase of the dipole becomes opposite from (**a**) to (**s**), reflecting to the SU(2) nature of orbital angular momentum upon the rotation in SO(3).

Next, we have also confirmed the pseudo-rotator operation for orbital angular momentum states, demonstrated previously for polarisation states. For polarisation, we just needed to rotate a HWP to realise a mirror reflection, which becomes a rotational operation for the input of horizontally polarised state. We have confirmed above that the pair of cylindrical lenses behave as the NOT operation, represented by $-i \sigma _{1}^{\textrm{O}}$, such that we can realise the pseudo-rotator, simply by rotating the pair of the cylindrical lenses, while keeping the faces with the fixed separation distance of 2*f*. Figure 5 confirms the expected counter-clock-wise rotation of the dipole images upon the rotation of the pair of the cylindrical lenses. In particular, upon the rotation of 45 $^{\circ }$ at the Fig. 5j, the horizontal dipole was mirror reflected to become the vertical dipole. The rotated NOT operation for this angle corresponds to $-i \sigma _{2}^{\textrm{O}}$, which means the $\pi$ rotation along the $L_2$ axis. Therefore, we have obtained both $\sigma _{1}^{\textrm{O}}$ and $\sigma _{2}^{\textrm{O}}$, except for the global phase, and we can also represent $\sigma _{3}^{\textrm{O}}$ by successive applications of $\sigma _{1}^{\textrm{O}}$ and $\sigma _{2}^{\textrm{O}}$ for orbital angular momentum, since $\sigma _{3}^{\textrm{O}}=i\sigma _{1}^{\textrm{O}} \sigma _{2}^{\textrm{O}}$ due to the commutation relationship. Here, it is important to be aware that the NOT operation for orbital angular momentum does not change the polarisation state at all. This means that we can prepare the single qubit operations of ${\bf{1}} \otimes \sigma _{1}$, ${\bf{1}} \otimes \sigma _{2}$, and ${\bf{1}} \otimes \sigma _{3}$ in Table 1. Therefore, the SU(2) operations are exclusively applicable to orbital angular momentum without changing the spin state. Similarly, we can apply an arbitrary single-qubit operation to the spin state without changing the orbital angular momentum state by using the Poincaré rotator.

### CNOT operation

Now, we are ready to discuss the CNOT operation for coherent photons with spin and orbital angular momentum (OAM) states. Table 3 shows the truth table for the CNOT operation. We need to keep OAM for horizontally polarised states, while we apply the NOT operation for vertically polarised states. We used the Polarisation-Beam-Splitter (PBS) to split the input beam into two beams with orthogonal polarisation states to realise the CNOT operation (Fig. 1d).

**Table 3:** Truth table of CNOT operation to SU(4) states of spin and orbital angular momentum (OAM).

Input states	Spin	OAM	Output states	Spin	OAM
	$\sigma$	*m*		$\sigma$	*m*
$|1\rangle = |\uparrow , \uparrow \rangle = |\textrm{H}, \textrm{L} \rangle$	1	1	$|1\rangle = |\uparrow , \uparrow \rangle = |\textrm{H}, \textrm{L} \rangle$	1	1
$|2\rangle = |\uparrow , \downarrow \rangle = |\textrm{H}, \textrm{R} \rangle$	1	-1	$|2\rangle = |\uparrow , \downarrow \rangle = |\textrm{H}, \textrm{R} \rangle$	1	-1
$|3\rangle = |\downarrow , \uparrow \rangle = |\textrm{V}, \textrm{L} \rangle$	-1	1	$|4\rangle = |\downarrow , \downarrow \rangle = |\textrm{V}, \textrm{R} \rangle$	-1	-1
$|4\rangle = |\downarrow , \downarrow \rangle = |\textrm{V}, \textrm{R} \rangle$	-1	-1	$|3\rangle = |\downarrow , \uparrow \rangle = |\textrm{V}, \textrm{L} \rangle$	-1	1

Previously, Lopes et al. achieved the CNOT operation by using a pentaprism^18^ rather than cylindrical lenses in our setup. The pentaprism preserves the handedness both for spin and orbital angular momentum upon two reflections by mirrors through the propagation, and the introduction of one pentaprism only for one of the arms of Mach-Zehnder interferometer allows the difference of parities for number of reflections by mirrors^18^. Here, we introduced two cylindrical lenses to change the handedness of orbital angular momentum only, while preserving the spin state as vertical polarisation during the NOT operation. On the other hand, we have not changed the handedness for horizontally polarised state, while adjusting the phase by several phase-plates to compensate the difference in path lengths.

**Fig. 6:**
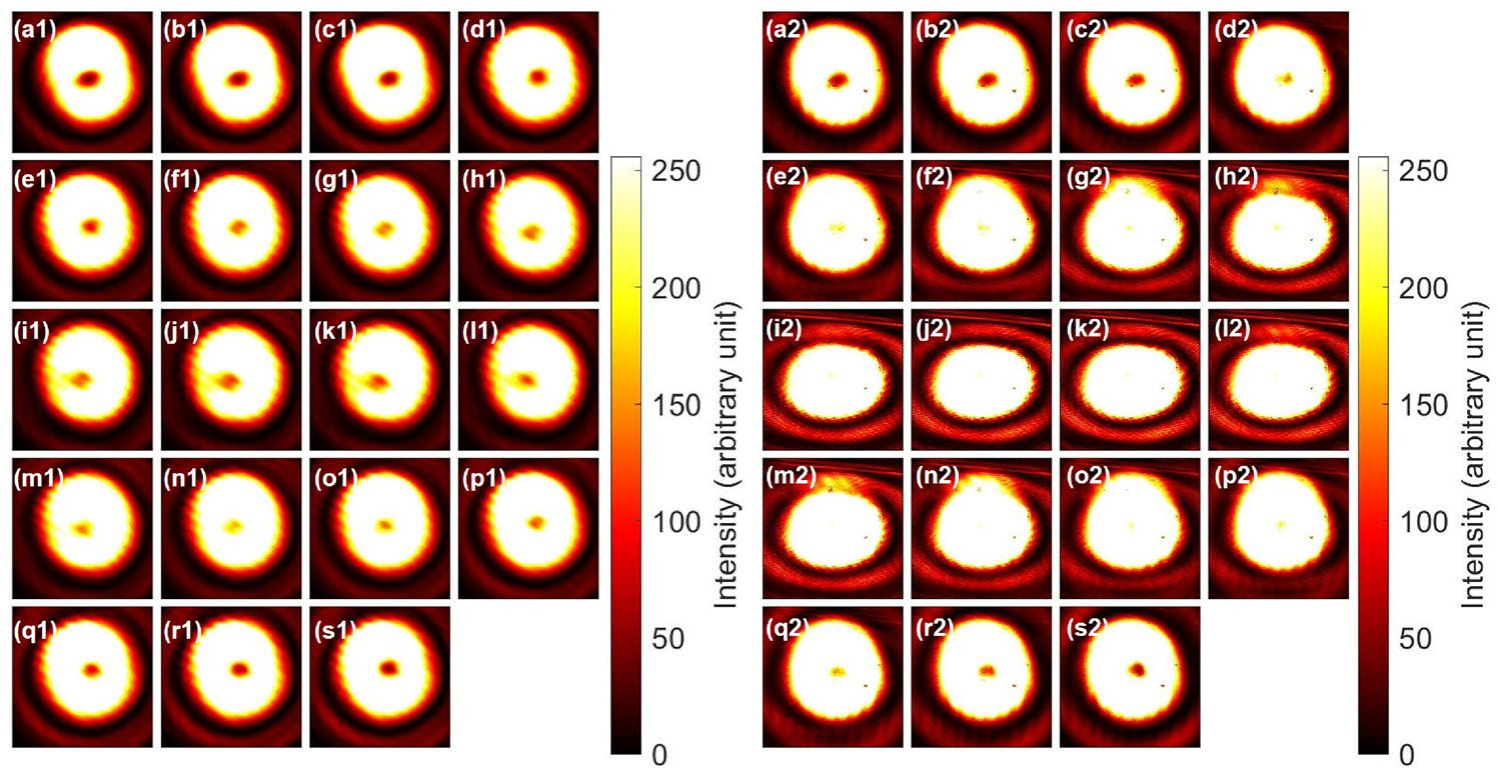
CNOT operations for spin and orbital angular momentum. Polarisers (PL2 and PL3) were not inserted. (**a1**)–(**s1**) Inputs of macroscopically entangled states, which were superposition states between $|\textrm{H} \rangle _{\textrm{S}}|\textrm{R} \rangle _{\textrm{O}}$ and $|\textrm{V} \rangle _{\textrm{S}}|\textrm{L} \rangle _{\textrm{O}}$, and HWP2 was rotated from 0 to 90 $^{\circ }$ with a step of 5 $^{\circ }$. (**a1**) is purely $|\textrm{H} \rangle _{\textrm{S}}|\textrm{R} \rangle _{\textrm{O}}$, while (j1) is purely $|\textrm{V} \rangle _{\textrm{S}}|\textrm{L} \rangle _{\textrm{O}}$. (**a2**)–(**s2**) Output far-field images after the CNOT operation. (**a2**) should be preserved to being $|\textrm{V} \rangle _{\textrm{S}}|\textrm{L} \rangle _{\textrm{O}}$. (**j2**) should be reverted for orbital angular momentum, such that the state must be $|\textrm{V} \rangle _{\textrm{S}}|\textrm{R} \rangle _{\textrm{O}}$.

Figure 6 shows the far-field images of inputs and outputs after the CNOT operations. We have rotated PL2 to change the direction of the projection for the polarisation states. Unfortunately, we cannot distinguish left and right vortices from images. The output images were also similar, since we could not distinguish the chiralities.

**Fig. 7:**
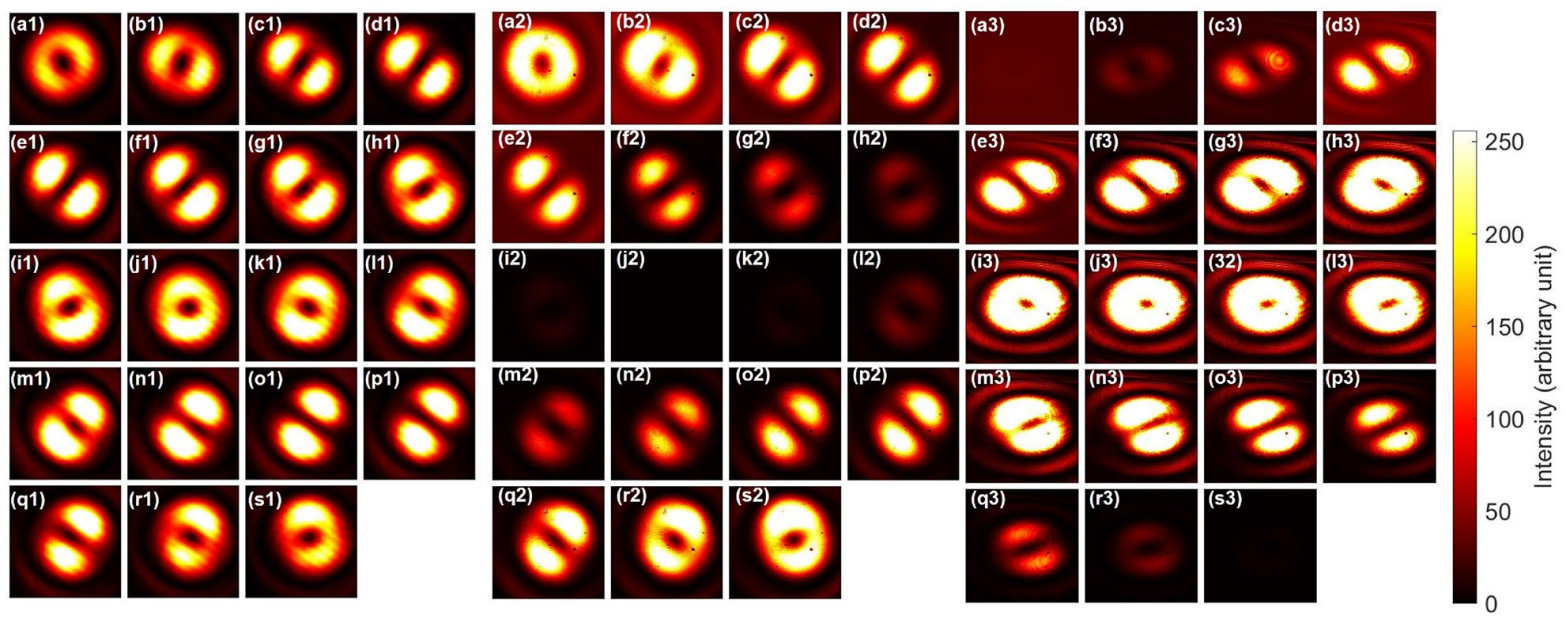
CNOT operations for the singlet state with spin and orbital angular momentum. (**a1**)–(**s1**) Input images were taken after rotating PL2 from 0 to 180 $^{\circ }$ with a step of 10 $^{\circ }$. This changes the direction of the Bell projection for polarisation states. At the diagonal polarisation of (**e1**), the dipole was aligned along the anti-diagonal direction, such that the phase was adjusted to be singlet. (**a2**)–(**s2**) Output far-field images for horizontal polarisation, projected by PL3, after the CNOT operation. (**e2**) was controlled to keep the anti-diagonal dipole. (**a3**)–(**s3**) Output far-field images for vertical polarisation, projected by PL3, after the CNOT operation. (**e3**) was rotated to be the diagonal dipole, which corresponds to the NOT operation. We can also confirm the expected CNOT operation for the input of (**n1**) with the controlled preservation in (**n2**), while the NOT operation worked properly for (**n3**).

Then, we have employed PL2 and PL3 to project the polarisation states to improve the visibilities (Fig. 7). The Bell projection of the coherent state^40^ works by selecting a particular polarisation state by the polariser. Suppose we have prepared the singlet state, $|\textrm{Singlet} \rangle =(|\uparrow ,\downarrow \rangle -|\downarrow ,\uparrow \rangle )/\sqrt{2} =(|\textrm{H}\rangle _{\textrm{S}}|\textrm{R}\rangle _{\textrm{O}}-|\textrm{V}\rangle _{\textrm{S}}|\textrm{L}\rangle _{\textrm{O}})/\sqrt{2}$, as an input state, the singlet state can be represented by diagonal (D) and anti-diagonal (A) basis as $|\textrm{Singlet} \rangle =(|\textrm{A}\rangle _{\textrm{S}}|\textrm{D}\rangle _{\textrm{O}}-|\textrm{D}\rangle _{\textrm{S}}|\textrm{A}\rangle _{\textrm{O}})/\sqrt{2}$. This means that the total angular momentum vanishes for the singlet state, regardless of the selection of the basis. The Bell projection corresponds to the selection of the particular polarisation state, which projects the orbital angular momentum state to be anti-diagonal dipole state. If the polariser is aligned to be the diagonal direction, the Bell projection works as $|\textrm{Singlet} \rangle \rightarrow -|\textrm{D}\rangle _{\textrm{S}}|\textrm{A}\rangle _{\textrm{O}}/\sqrt{2}$ (Fig. 7(e1)). Similarly, if the polariser is aligned to be the anti-diagonal direction, the Bell projection works as $|\textrm{Singlet} \rangle \rightarrow |\textrm{A}\rangle _{\textrm{S}}|\textrm{D}\rangle _{\textrm{O}}/\sqrt{2}$ (Fig. 7(n1)).

The input images were obtained after rotating PL2 continuously for the classical entangled photons (Fig. 7). For example, Fig. 7(e1) shows the anti-diagonal dipole with the diagonal polarisation, which means the entangled state was indeed singlet to have zero overall angular momentum. The corresponding output image for the horizontally polarised state is shown in Fig. 7(e2), which shows that the direction of the anti-diagonal dipole was controlled to be preserved. On the other hand, for the vertically polarised state, the beam must be passing though the NOT operator of HWPS, and in fact, the dipole was reversed to be pointing to the diagonal direction, as shown in Fig. 7 (e3). This clearly shows the successful CNOT operation for coherent photons with spin and orbital angular momentum. The similar CNOT operation could be confirmed for the input of Fig. 7(n1) to become the output of Fig. 7(n2) for controlled operation, while the NOT operation was successfully applied to Fig. 7(n3) for vertical polarisation.

**Fig. 8:**
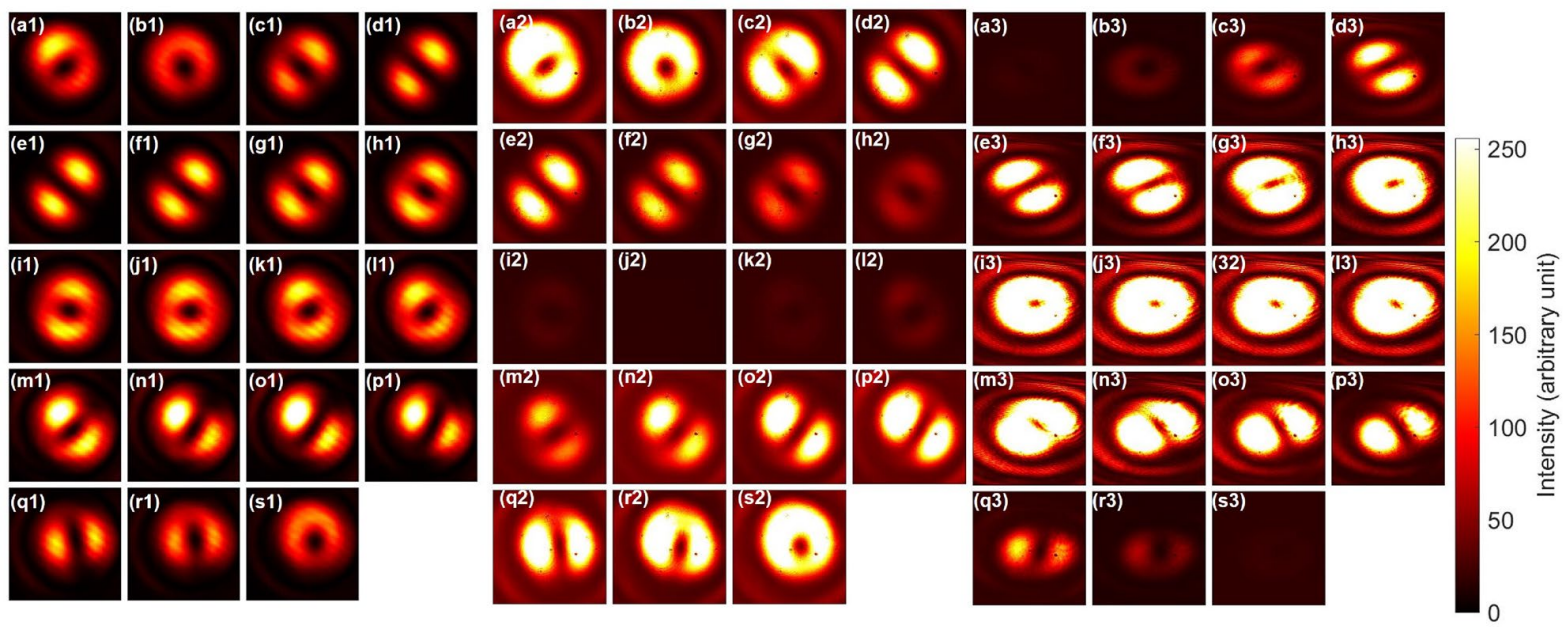
CNOT operations for the triplet state with spin and orbital angular momentum. (**a1**)–(**s1**) Input images were taken after rotating PL2 from 0 to 180 $^{\circ }$ with a step of 10 $^{\circ }$. At the diagonal polarisation of (**e1**), the dipole was aligned along the diagonal direction, such that the phase was adjusted to be triplet. (**a2**)–(**s2**) Output far-field images for horizontal polarisation, projected by PL3, after the CNOT operation. (**e2**) was controlled to keep the diagonal dipole. (**a3**)–(**s3**) Output far-field images for vertical polarisation, projected by PL3, after the CNOT operation. (**e3**) was rotated to be the anti-diagonal dipole, which corresponds to the NOT operation. We can also confirm the expected CNOT operation for the input of (**n1**) with the controlled preservation in (**n2**), while the NOT operation worked properly for (**n3**).

Simiraly, we have also examined that the CNOT operation works properly for triplet states (Fig. 8). The triplet state is given by $|\textrm{Triplet} \rangle =(|\uparrow ,\downarrow \rangle +|\downarrow ,\uparrow \rangle )/\sqrt{2} =(|\textrm{H}\rangle _{\textrm{S}}|\textrm{R}\rangle _{\textrm{O}}+|\textrm{V}\rangle _{\textrm{S}}|\textrm{L}\rangle _{\textrm{O}})/\sqrt{2}$, which can be rewritten as $|\textrm{Triplet} \rangle =(|\textrm{D}\rangle _{\textrm{S}}|\textrm{D}\rangle _{\textrm{O}}-|\textrm{A}\rangle _{\textrm{S}}|\textrm{A}\rangle _{\textrm{O}})/\sqrt{2}$. Therefore, the Bell projection along the diagonal direction by the polariser corresponds to $|\textrm{Triplet} \rangle \rightarrow |\textrm{D}\rangle _{\textrm{S}}|\textrm{D}\rangle _{\textrm{O}}/\sqrt{2}$ (Fig. 8(e1)). The diagonal dipole is preserved for the horizontal polarisation (Fig. 8(e2)), while it is inverted for the vertical polarisation (Fig. 8(e3)) after the CNOT operation. The proper CNOT operations both for singlet (Fig. 7) and triplet (Fig. 8) states guarantee that the phase is properly maintained upon the CNOT operations.

**Fig. 9:**
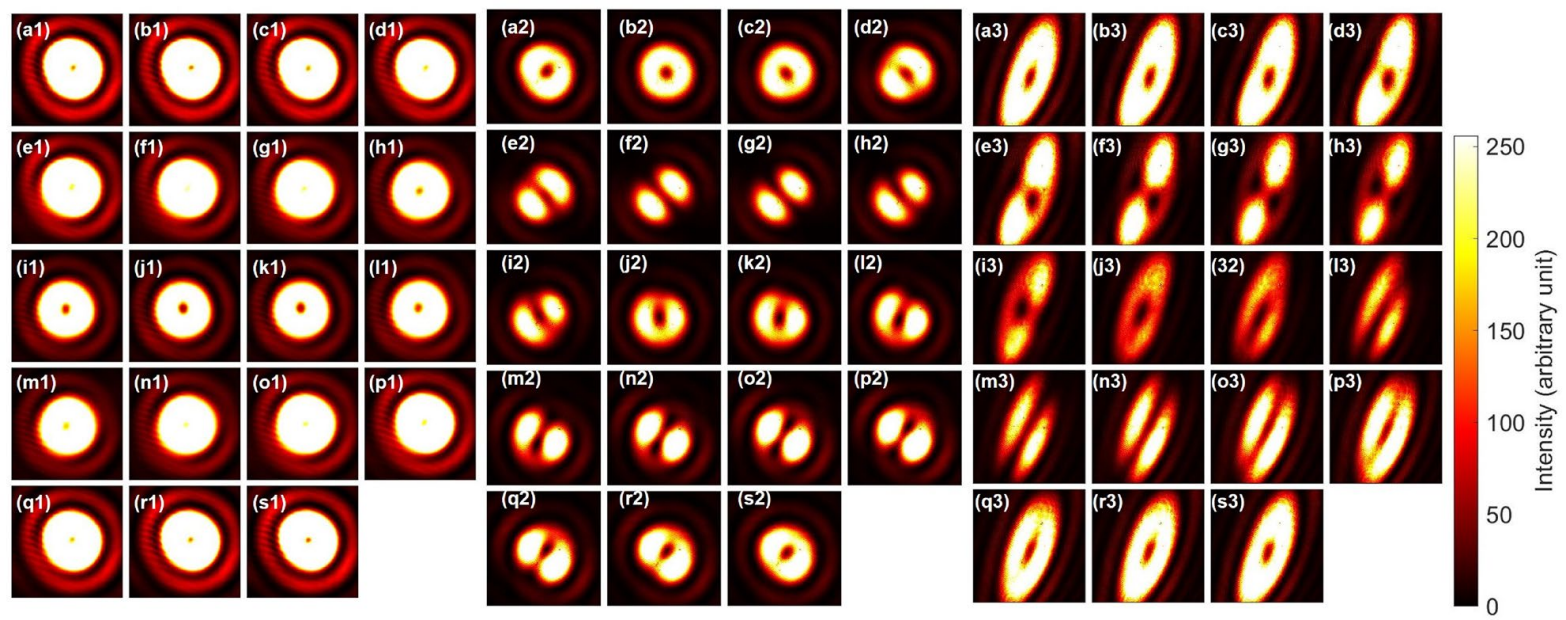
CNOT operations for spin and orbital angular momentum, obtained from the inputs, made of diagonally and anti-diagonally polarised states. PL2 was not employed in this measurement. (**a1**)–(**s1**) Inputs of macroscopically entangled states, which were superposition states between $|\textrm{D} \rangle _{\textrm{S}}|\textrm{R} \rangle _{\textrm{O}}$ and $|\textrm{A} \rangle _{\textrm{S}}|\textrm{L} \rangle _{\textrm{O}}$. Input images were taken after rotating HWP2 from 0 to 90 $^{\circ }$ with a step of 5 $^{\circ }$. (**e1**) was made of the sum of these states. (**a2**)–(**s2**) Output far-field images for horizontal polarisation, projected by PL3, after the CNOT operation. (**e2**) was controlled to keep the diagonal dipole. (**a3**)–(**s3**) Output far-field images for vertical polarisation, projected by PL3, after the CNOT operation. The input for (**e3**) is anti-diagonal dipole, which was successfully reverted to be the diagonal dipole.

We have also confirmed the CNOT operation by preparing input using diagonal and anti-diagonal polarisation states (Fig. 9). Here, we used HWP5 and HWP6 to convert the horizontally and vertically polarised states to be diagonal and anti-diagonal polarisation states, respectively (Method). This was achieved simply by rotating HWP5 and HWP6 with +22.5 $^{\circ }$ and -22.5 $^{\circ }$, respectively. In this way, we have removed PL2, such that we have not employed the Bell projection in this set-up. Then, the output states from the generator becomes the superposition state between $|\textrm{D} \rangle _{\textrm{S}}|\textrm{R} \rangle _{\textrm{O}}$ and $|\textrm{A} \rangle _{\textrm{S}}|\textrm{L} \rangle _{\textrm{O}}$. Suppose the amplitudes for these states were the same, for example, and the input state becomes $(|\textrm{D} \rangle _{\textrm{S}}|\textrm{R} \rangle _{\textrm{O}}+i|\textrm{A} \rangle _{\textrm{S}}|\textrm{L} \rangle _{\textrm{O}})/\sqrt{2}$ at Fig. 8(e1), upon adjusting the phase by HWP4. If we use HV bases for polarisation, this state corresponds to be $|\textrm{H} \rangle _{\textrm{S}}(|\textrm{R} \rangle _{\textrm{O}}-i|\textrm{L} \rangle _{\textrm{O}})/2 +|\textrm{V} \rangle _{\textrm{S}}(|\textrm{R} \rangle _{\textrm{O}}+i|\textrm{L} \rangle _{\textrm{O}})/2 = ( |\textrm{H} \rangle _{\textrm{S}}|\textrm{D} \rangle _{\textrm{O}} +i |\textrm{V} \rangle _{\textrm{S}}|\textrm{A} \rangle _{\textrm{O}} )/\sqrt{2}$, which means that we can insert the diagonal dipole to the horizontal input and it was preserved at the output of Fig. 9 (e2). On the other hand the anti-diagonal dipole was inserted into the vertical input, and it was inverted upon the CNOT operation to become the diagonal dipole at the output, shown in Fig. 9(e3). Consequently, both dipoles for horizontal and vertical polarisation states are aligned to the same direction after the CNOT operation.

## Conclusion

We have developed a representation theory for coherent photons, which shows the quantum mechanical superposition states can be realised for macroscopic number of photons with rotational SU(*N*) symmetry. We have shown both spin and orbital angular momentum can form SU(2) states to represent expectation values of generators of rotations in SO(3) Poincaré spheres. By extending the orthogonal states to SU(4), we have shown that the arbitrary superposition states could be realised by adjusting phases and amplitudes of basis states. As an application of our theoretical formulation, we have experimentally demonstrated CNOT operations to coherent photons, which were visualised by taking far-field images upon projections by polarisers.

Finally, we would like to discuss opportunities and limitations of classical entanglement of coherent photons^8–18^. We believe there exists a practical advantage to use classical entanglement of coherent photons compared with single photons^36,47,49–52^, since experiments are much easier to handle due to macroscopic number of photons involved. The principle behind the classical entanglement is the SU(4) symmetry of coherent photons and non-separability of spin and orbital angular momentum states. The classical entanglement will be suitable for applications in sensing, metrology, communications, and computation, which require small number of qubits^16^. Here, it is important to be aware that the classical entanglement is mostly local entity with a few exceptions by spatial mode controls^15,53^, such that we do not expect non-local correlation over long distance realised in quantum entanglement^36,47,49–52^. In order to scale our scheme from classical to quantum entanglement, it is inevitable to reduce the power down to the single photon level. Consequently, we must reduce the loss significantly to realise high fidelities required for various applications such as quantum computing^40^. This is a significant engineering challenge, and a lot of researchers are working for single photons to realise fault-tolerant quantum computing^36,47,49–52^. The use of OAM would help to increase the internal degrees of freedom, but it would be a significant engineering challenge to allow the precise control of phases and amplitudes in a single photon level. We need to use single photon sources and realise nearly perfect alignments without increasing the loss. Silicon photonic integration circuits would help the reproducibility and the precise operation^47^, while the coupling to optical fibres is also a challenge. On the other hand, we are more favourable to use coherent photons as classical entanglement with higher order symmetry of SU(4) or above^54–58^. It is expected to realise various topological structured lights^59,60^ by manipulating coherent photons with SU(*N*) degrees of freedom. The higher order symmetry of coherent photons will expand the bandwidths of data recording and data transmission^59^. It is also expected to explore the coherent photons as a platform to examine various concepts like optical skyrmions^59,61^, photonic quantum-chromo-dynamics (QCD), and photonic Dirac cones.

## Methods

We used a Diode-Pumped Solid State (DPSS) Laser-Diode (LD) at the wavelength of 532nm, operated at the temperature of 20 $^{\circ }$C with the constant current of 150 mA (Fig. 1). The output power was about 10mW. We used a Collimator Lens (CL) of the focal length (*f*) of 100.0mm. The beam was refined by a Pin Hole (PH) with the diameter of 200 ${ \upmu }$m for a Gaussian profile. We also introduced a Polariser (PL) to make the input beam to be horizontally polarised. We used several Half-Wave-Plates (HWPs), Quarter-Wave-Plates (QWPs), and mirrors (M) to control the phases and the amplitudes of the input beam. The details of the proposed Poincaré rotator (PR) to control the polarisation state appear elsewhere. We used a Polarisation-Beam-Splitter (PBS) to split horizontally and vertically polarised components into 2 beams, whose relative amplitudes were controlled by rotating the HWP2, while the fast axis of HWP1 is fixed to be horizontal. The phase of vertically polarised beam was controlled by a series of operations through QWP1, HWP3, HWP4,and QWP2. Here, it is important to convert the spin state of polarisation to the orbital state by vortex lenses (VLs)^17,24,25,27,30,46,62–66^. The VL^27^ is also known as the spiral phase plate, which is made of a dielectric material with spiral thickness variation, allowing to induce the azimuthal dependent phase shift. The advantage to use the VL for our application is the polarisation independence, while the *q*-plate^67^ converts spin to orbital angular momentum, and thus, the *q*-plate is polarisation dependent. The VL can change the chirality of the generated vortex by flip-flop exchange, although we used the same orientation for VL1 and VL2 to generate left vortices. In this case, the horizontally polarised Gaussian beam is inserted into VL1 to generate a left vortex, while keeping horizontal polarisation, and reflected by the mirror of the Non-Polarisation-Beam-Splitter (NPBS) to change its chilarity to a right vortex. Therefore, the right vortex with horizontal polarisation is generated after the NPBS. In the opposite arm, vertically polarised beam is inserted into VL2 to generate a left vortex, whose state will not be change to passing through the NPBC. In this way, spin and orbital angular momentum states are entangled to form a collimated beam with internal structures.

The output beam from the macroscopic entanglement generator has a doughnut profile, and thus it is difficult to distinguish the chirality of the twisted state from far-field images. This issue was overcome by inserting PL2 to achieve the Bell projection. For example, by setting PL2 to pass only for diagonally polarised state, the left and the right vortices are superimposed to form a dipole image in the far-field. The orientation of the dipole depends on the relative phase between the left and the right vortices (Fig. 1c), which can be controlled by rotating HWP4.

Alternatively, we can also change the polarisation states from the horizontal polarisation to the diagonal polarisation in the arm with M1 by HWP5, while the polarisation along the M2 is rotated to the anti-diagonal polarisation by HWP6. This allows to realise a dipole shape in a far-field image for horizontal and vertical polarisation. The polarisation state and the input of the image was monitored by a polarimeter (PM) and a CMOS camera (CMOS1). It is an advantage to use coherent photons, that we can split some of the beam to monitor states without measuring the entire beam.

The centre of the CNOT operation consists of the PBS and Cylindrical lenses (Cyl). We used Cyl1 and Cyl2 with $f=20.0$ mm, which are placed with the distance of 2*f* (Fig. 1b). This allows us to realise the Half-Wave Phase-Shift (HWPS) for twisted states without changing the polarisation state^48^ . The HWPS corresponds to change the left vortex to the right vortex and *vice versa* (the red curve of Fig. 1c). This will also change the diagonally twisted dipole to the anti-diagonally twisted dipole, and vice versa. Thefore, the HWPS works as a NOT operator for twisted photons. By using the NOT operation only for the vertically polarised state, while the horizontally polarised state is preserved, we can realise the CNOT operation. We have also controlled the phase of the horizontally polarised states by using the series of operations by QWP2, HWP5, HWP6, QWP4, and HWP7 for adjusting the difference of path lengths. PL3 was optionally used for taking images after the polarisation, and the final far-field image was taken by a camera (CMOS2).

## Data Availability

The data that support the findings of this study are available from the author upon reasonable requests.
